# Circular RNA CCT3 is a unique molecular marker in bladder cancer

**DOI:** 10.1186/s12885-023-11510-0

**Published:** 2023-10-13

**Authors:** Lin Luo, Qingzhi Xie, Yunchou Wu, Ping Li, FuQiang Qin, Dunming Liao, KangNing Wang

**Affiliations:** 1 Department of urology surgery, The First Affiliated Hospital of Shaoyang University, No. 39, Tongheng Street, Shuangqing District, Shaoyang City, Hunan Province 422000 China; 2 Department of urology surgery, Xiangya Hospital Central South University, Changsha City, Hunan Province 410008 China

**Keywords:** Circular RNA CCT3, Bladder cancer, Diagnosis, Prognosis, PP2A, miR-135a-5p, Molecular marker

## Abstract

This study surveyed circular RNA CCT3 in bladder cancer (BCa). We recruited 85 BCa patients and 40 normal controls (Normal) and collected clinical specimens for analysis. circRNA CCT3 expression was analyzed by RT-qPCR, diagnostic accuracy was calculated by ROC curves, and survival outcomes were evaluated by survival curves. CircRNA CCT3 was overexpressed or knocked down in cells, thereafter to observe the changes in cell malignant phenotypes. The downstream molecules of circRNA CCT3 were detected. Our data suggest that circRNA CCT3 was upregulated in human BCa and was associated with poor survival outcomes of BCa patients. In cell experiments, overexpressing circRNA CCT3 promoted BCa cell malignancy, whereas silencing circRNA CCT3 did the opposite. In addition, circRNA CCT3 modulated PP2A expression by miR-135a-5p. This study demonstrates that circRNA CCT3 is a diagnostic and prognostic biomarker in BCa patients and is a tumor promoter in BCa.

**Supplementary Information:**

The online version contains supplementary material available at 10.1186/s12885-023-11510-0.

## Introduction

The most common subtype of bladder cancer (BCa) is urothelial carcinoma or transitional cell carcinoma, accounting for more than 90% of all cases [1]. In 2020, BCa ranked 9th in incidence globally and 13th in cancer-related deaths among all cancers [2]. BCa can be divided into two groups according to their different behaviors: high-grade muscle-invasive BCa (MIBC) and low-grade non-muscle-invasive BCa. Surgery is the main treatment for BCa. However, the advanced disease often progresses to MIBC for which the treatment is often suboptimal [3, 4]. This is because MIBC often metastasizes to lymph nodes and distant organs, resulting in an extremely poor prognosis and low survival rate [5]. In addition, advanced BCa is less sensitive to chemoradiotherapy [3]. Therefore, early diagnosis of BCa and the search for reliable biomarkers and effective therapeutic targets are particularly important.

CircRNAs are non-coding RNAs with high stability [6]and serve greatly in disease pathogenesis [7, 8]. Reports have confirmed that circRNAs are closely related to BCa development [9–12]. For example, circSOBP inhibits BCa proliferative and metastatic activities. In addition, circMGA mediates CD8 T cell infiltration and immunotherapy in BCa [13]. circTAF4B is oncogenic regarding to BCa cell growth, metastasis and EMT [14]. High expression of circ-SMARCA5 is related to advanced tumor characteristics and poor survival in BCa patients [15]. Hsa_circ_0004680 is a newly discovered circRNA whose host gene is CCT3, so it is named circRNA CCT3. circRNA CCT3 acts as an oncogene in colorectal cancer [16] and multiple myeloma [17]. However, whether circRNA CCT3 can act as a unique molecular marker for BCa and influence the progression of BC has not yet been demonstrated.

It is well known that the main function of circRNA is to function by binding to miRNA [18] Based on bioinformatic analyses, miR-135a-5p was identified as binding to circRNA CCT3. miR-135a-5p acts as a cancer suppressor in a variety of cancers, such as non-small cell lung cancer [19] and breast cancer [20]. We found the binding sequence of miR-135a-5p in the protein phosphatase 2 A (PP2A) 3’UTR through bioinformatic analyses. PP2A has been implicated in many cancer types including BCa [21–23]. We speculated that circRNA CCT3 mediates BCa cell progression and acts as a potential regulator of PP2A and aimed to provide new perspectives on BCa pathogenesis and a new theoretical basis for the targeted therapy of BCa.

## Materials and methods

### Clinical sample

From December 2014 to May 2018, urine samples from 85 patients with BCa and 40 healthy subjects were obtained from The First Affiliated Hospital of Shaoyang University. These urine samples were taken from the subjects’ morning urine. Urine was collected in a sterile environment and that only midstream urine was stored for analysis. For each patient, 25 mL of urine was collected for analysis. The urine was quickly stored at -80 °C to avoid RNA degradation. All clinical experiments were conducted following the principles of The First Affiliated Hospital of Shaoyang University Ethics Committee. Written consent has been obtained from all subjects.

### Patient’s information

Clinicopathological data included age, gender, multiple, tumor size, tumor stage (Union for International Cancer Control stages), lymph node metastasis, and pathological grade (2004 World Health Organization urothelial tumor classification) were obtained. All patients were followed-up until April 30, 2020, during which the recurrence-free survival (RFS) and overall survival (OS) were calculated [15].

### Microarray analysis

Human CircRNA Array v2.1 (CapitalBio Technology, China) performed CircRNA microarray analysis. After RNA quantification using NanoDrop ND-1000, total RNA was digested with RNase R (Epicentrtechnologies, USA), amplified by Arraystar Super RNA Labeling Kit and transcribed. circRNAs were hybridized to Arraystar Human circRNA Array V2 (8 × 15 K, Arraystar), scanned with an Agilent G2505C, and analyzed by Agilent Feature Extraction software 1.0.1.1. R. Fold Change filtering identified differentially expressed circRNAs and hierarchical clustering revealed distinguishable circRNA expression patterns.

### Cell transfection

SV-HUC-1, human bladder epithelial SV40 immortalized cell line (ATCC® CRL-9520) and BCa cell line T24 (ATCC® HTB-4) were allowed to stand in F-12 K medium (No. 30-2004) and McCoy’s 5a Modified Medium (No. 30-2007), respectively. Both the media (ATCC) was supplemented with 10% FBS.

circRNA CCT3 overexpression and low expression plasmids (circRNA CCT3 and si-circRNA CCT3) were provided by GeneTop (Changsha, China) and transfected using Lipofectamine™ 3000 (Thermo Fisher Scientific, USA) [24].

### CCK-8 assay

Cells (3 × 10^4^/well) were mixed with 10 µL of CCK-8 solution (Dojindo) at 24, 48, and 72 h, respectively. After 2 h, the absorbance (450 nm) was measured with a microplate reader (Bio-Rad).

### Colony formation experiment

Cells (6000 cells/well) were maintained in 10% FBS-DMEM for 8 d and the number of colonies was counted after 0.1% crystal violet staining [10].

### Migration and invasion analysis

Cells (1 × 10^5^) were suspended in 200 µl of serum-free medium and transferred into the upper chamber of transwell (8 μm, Costar), whose bottom chamber contained 10% FBS medium. After 2 h, lower surface cells after 0.1% crystal violet staining were seen under a microscope (Olympus, Japan). Matrigel (BD Biosciences) was only utilized in invasion analysis [8].

### Flow cytometry

Cells resuspended in 600 µl of binding buffer were stained with Annexin V/FITC (5 µl) and propidium iodide (5 µl). Results were analyzed using FlowJo 7.6 software.

### RNA extraction and analysis

Total RNA was extracted by Trizol (15596-018, Invitrogen) and reverse transcribed into cDNA by Primescript™ RT reagent (RRO37A, TaKaRa) or Mir-X miRNA First Strand Synthesis Kit (638,315, Clontech, USA). PCR was implemented with SYBR Premix Ex TaqTM II Kit (TaKaRa) in an ABI PRISM 7300 system. U6 and GAPDH served as internal controls. The primers shown in Table 1 were synthesized by Bio Just (Wuhan, China). 2^−ΔΔCt^ was the gene calculation formula [25].

**Table 1:** Primer sequences in PCR

Gene	Primers for PCR (5’-3’)	
circRNA CCT3	Forward	AATTAGCCGGACCCAGGATG
	Reverse	ACAATGCCTCCCATTGGGTC
miR-135a-5p	Forward	CCAGGCTTCCAGTACCATTAGG
	Reverse	GTTTCCGAGAGAGGCAGGTG
PP2A	Forward	TTGATCGCCTACAAGAAGTTCCCCA
	Reverse	CTCTACGAGGTGCTGGGTCAAACTG
U6	Forward	CTCGCTTCGGCAGCACA
	Reverse	AACGCTTCACGAATTTGCGT
GAPDH	Forward	GATGATCTT GAGGCTGTTGTC
	Reverse	CAGGGCTGCTTTTAACTCTG

Note: miR-135a-5p, microRNA-135a-5p; PP2A, Protein phosphatase 2 A; GAPDH, glyceraldehyde-3-phosphate dehydrogenase

### Immunoblotting

Proteins were prepared with RIPA lysis buffer (Beyotime, China), separated by 10% SDS-PAGE and transferred to PVDF membranes (Millipore). Then, the membrane was blocked with skim milk, hybridized with the primary antibodies (PP2A [1:1000, 05-421, MilliporeSigma], GAPDH [1:1000, 2118, CST]) and the secondary antibody (1:10000, ab6721, Abcam). Blots were analyzed with Quantity One software (Bio-Rad) after interaction with ECL reagent (Invitrogen) [26].

### RNA immunoprecipitation (RIP)

T24 cells were lysed using complete RIP lysis buffer (Millipore) and mixed with beads pre-coated with anti-Ago2 (Millipore) or anti-IgG (Millipore). At last, samples were subjected to gene RNA expression after proteinase K digestion (Invitrogen) [27].

### Luciferase reporter gene assay

circRNA CCT3 (circRNA CCT3-wt, circRNA CCT3-mut) and PP2A (PP2A-wt, PP2A-mut) containing wild-type (wt) and mutant (mut) miR-135a-5p binding sites were cloned into pmirGLO-Report luciferase vector (Promega, USA) and co-transfected with NC mimic or miR-135a-5p mimic by Lipofectamine 3000 (Invitrogen). A dual-luciferase assay system (Promega) was applied to the result analysis.

### Statistical analysis

All data were presented as mean ± standard deviation and evaluated by SPSS 20.0. Two sets of data were assessed by paired Student’s t-test or unpaired Student’s t-test while multiple sets of data were by ANOVA and Dunnett’s test. Chi-square test compared the categorical data, and ROC curve evaluated the diagnostic accuracy. Kaplan-Meier curve and log-rank test assessed the OS and RFS in patients with BCa, and Cox regression proportional hazard analysis examined prognostic factors. *P* < 0.05 represented significant differences.

## Results

### Baseline characteristics of BCa patients

BCa patients (mean age: 61.8 ± 10.5 years) included 36 women (23.1%) and 120 men (76.9%) (Table 2). The tumor was single in 106 cases (67.9%) and multiple in 50 cases (32.1%). The mean tumor size was 2.5–1.2 cm, with 108 cases (69.2%) in Ta-T1 stages, and 48 cases (30.8%) in T2-T4 stages. In addition, 13 cases (8.3%) developed LNM; 96 cases (61.5%) were in low pathological grade and 60 cases (38.5%) were in high pathological grade.

**Table 2:** Baseline characteristics of bladder cancer patients

Items	Bladder cancer patients (n = 85)
Age (years),mean ± SD	62.5 ± 7.5
Gender, No.(%)	
Female	27 (31.8%)
Male	58 (68.2%)
Multiplicity, No.(%)	
Single	54 (63.5%)
Multiple	31 (36.5%)
Tumor size (cm), mean ± SD	2.4 ± 1.5
Tumor stage, No.(%)	
Ta&T1	51 (60.0%)
T2-T4	34 (40.0%)
Lymph node metastasis, No.(%)	
No	65 (76.5%)
Yes	20 (23.5%)
Pathological grade, No.(%)	
Low	54 (63.5%)
High	31 (35.5%)

Note: SD, standard deviation

### circRNA CCT3 is highly expressed in BCa

**Fig. 1:**
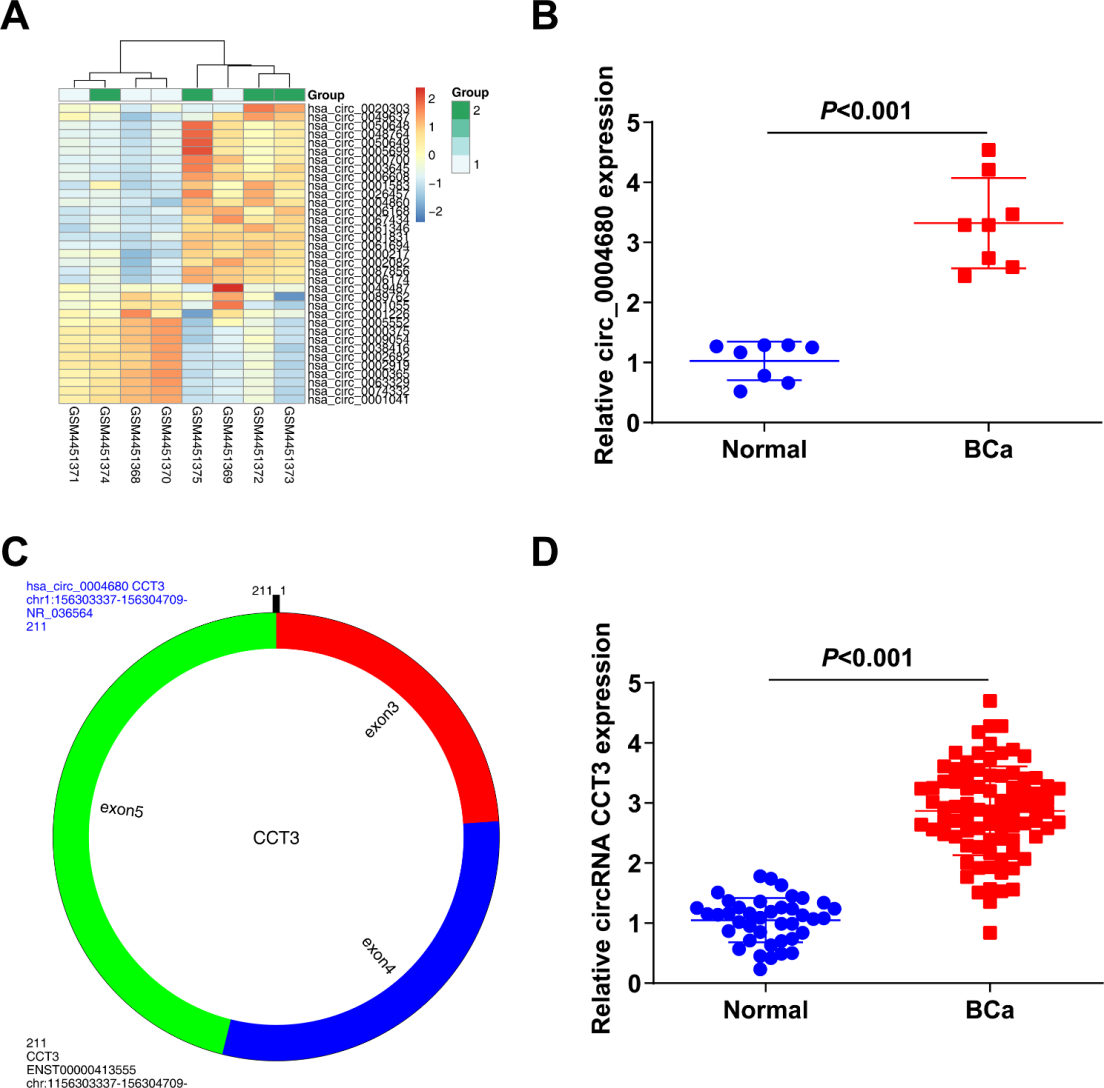
circRNA CCT3 is highly expressed in BCa. **(A)** Cluster heat maps showing up- and down-regulated circRNAs in urine samples from BCa. Red indicates high expression and blue indicates low expression. **(B)** Relative expression of circ_0004680 in 8 normal controls and 8 BCa samples. **C**. The schematic diagram showing the production of hsa_circ_0004680. **D**. circRNA CCT3 expression in 40 normal urine samples and 85 urine samples with BCa.

To discover potential BCa-related circRNAs, urine samples from 8 healthy controls and 8 BCa patients were analyzed by microarray, and 21 circRNAs were upregulated and 14 were downregulated in BCa patients (Fig. 1A). Among the most significantly changed circRNAs, hsa_circ_0004680 was further filtered (Fig. 1B). hsa_circ_0004680 is located at chr1:156303337–156,304,709, and the host gene is CCT3, with 1372 bp genome length and 211 bp spliced length (Fig. 1C). To further evaluate the potential significance of circRNA CCT3, its expression was checked in 40 cases of healthy controls and 85 cases of BCa samples. It was discovered that circRNA CCT3 expression was elevated in BCa patients (Fig. 1D), suggesting that circRNA CCT3 is highly expressed in BCa and may be associated with tumor development.

### Diagnostic value of circRNA CCT3 in BCa patients

**Fig. 2:**
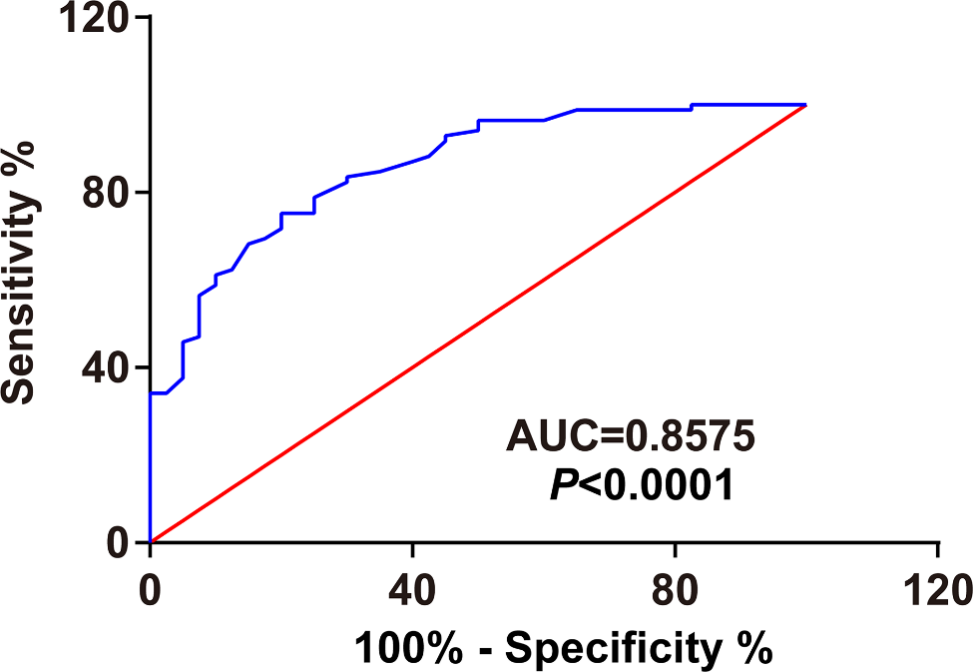
Diagnostic value of circRNA CCT3 in BCa patients. ROC curve showed the discriminative ability of plasma circRNA CCT3 in BCa patients and normal controls

To evaluate the diagnostic value of circRNA CCT3 in BCa patients, ROC analysis was employed to analyze the role of plasma circRNA CCT3 in distinguishing BCa patients from normal controls. The data presented that the optimal diagnostic cutoff value of circRNA CCT3 was 1.09, and the AUC value was 0.8575 (95% CI, 0.734–0.896) (Fig. 2), the sensitivity was 79.5% and specificity was 65.6% (Table 3). This suggests that circRNA CCT3 in plasma has potential diagnostic value for BCa.

**Table 3:** ROC curve analysis of the diagnostic value of plasma circRNA CCT3 in bladder cancer

Group	AUC (95% Confidence Interval)	P value	Sensitivity	Specificity
BCa vs. Normal	0.8575 (0.7897–0.9253)	< 0.0001	87.06	60

### Prognostic value of circRNA CCT3 in BCa patients

**Fig. 3:**
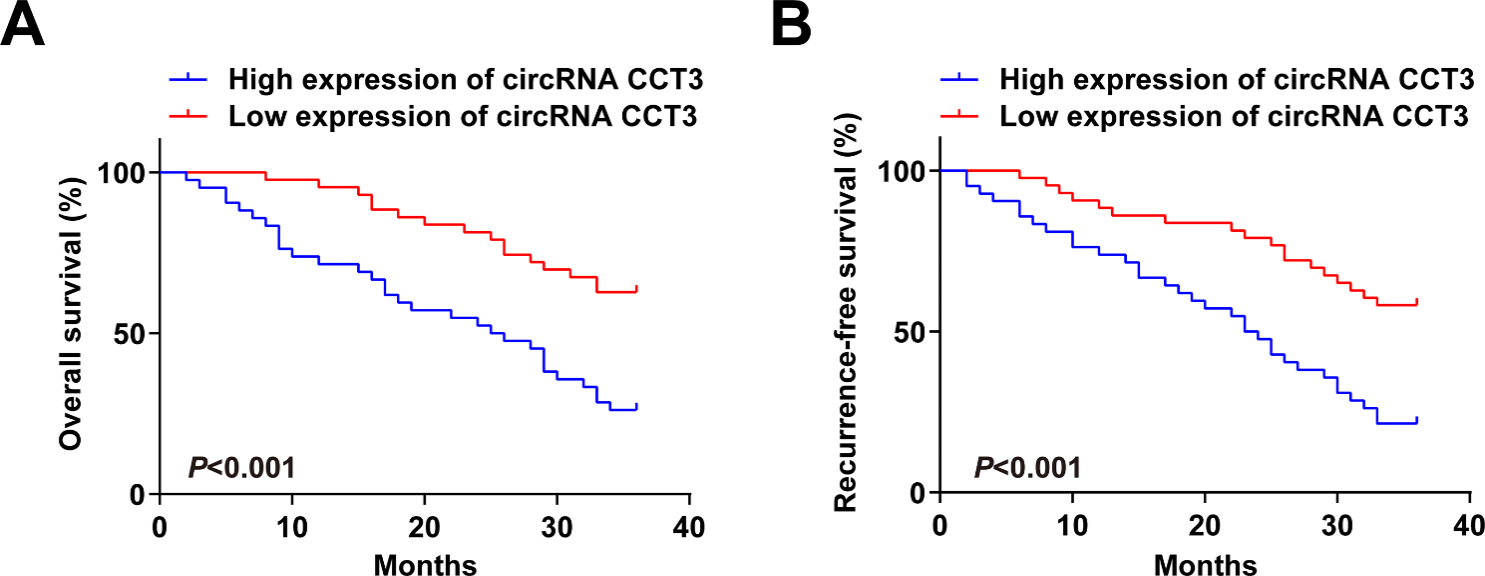
Prognostic value of circRNA CCT3 in BCa patients. **(A)** Overall survival and **(B)** recurrence-free survival **(B)** of BCa patients stratified by circRNA CCT3.

It was demonstrated in Kaplan-Meier analysis that circRNA CCT3 high expression was associated with poor OS (Fig. 3A; *P* = 0.005) and RFS (Fig. 3B; *P* = 0.002). COX regression analysis (Tables 4 and 5) discovered that circRNA CCT3 high expression (HR: 1.74, 95% CI: 1.31–2.01, P = 0.002), T stage (HR: 1.81, 95% CI: 1.51–2.13, P = 0.001), and tumor grade (HR: 2.02, 95% CI: 1.32–2.85, *P* = 0.028) were independent indicators for poor OS in BCa patient, while circRNA CCT3 high expression (HR: 1.95, 95% CI: 1.20–2.64, *P* = 0.001), T stage (HR: 2.11, 95% CI: 1.21–3.05, *P* = 0.001), and tumor grade (HR: 2.52, 95% CI: 1.16–3.31, *P* = 0.035) were those for poor RFS. These data suggest that circRNA CCT3 is an independent favorable prognostic factor in BCa patients.

**Table 4:** Univariate and multivariate analysis of prognostic factors associated with overall survival

Variables	Univaiate		Multivariate	
	HR (95% CI)	P-value	HR (95% CI)	P-value
Age(≥ 60 vs. 60)	1.07 (0.78–1.62)	0.158		
Gender (male vs. female)	1.05 (0.73–1.68)	0.356		
Multiplity (Single vs. Multiple)	1.27 (0.84–1.82)	0.129		
Tumor stage (T2-T4 vs. Ta-T1)	1.96 (1.65–2.28)	< 0.001*	1.82 (1.53–2.1)	0.002*
Lymph node metastasis (yes or no)	1.12 (0.78–1.87)	0.121		
Pathological grade (high vs. low)	1.54 (0.88–2.3)	0.092		
circRNA CCT3 expression (high vs. low)	1.83 (1.45–2.09)	0.001*	1.72 (1.28–2.03)	0.002*

**Table 5:** Univariate and multivariate analysis of prognostic factors associated with recurrence-free survival

Variables	Univaiate		Multivariate	
	HR (95% CI)	P-value	HR (95% CI)	P-value
Age (≥ 60 vs. 60)	1.17 (0.79–1.72)	0.246		
Gender (male vs. female)	1.15 (0.70–1.63)	0.168		
Multiplity (Single vs. Multiple)	1.17 (0.89–1.42)	0.629		
Tumor stage (T2-T4 vs. Ta-T1)	2.66 (1.35–3.48)	< 0.001*	2.12 (1.23–3.07)	0.001*
Lymph node metastasis (yes or no)	1.32(0.70–1.97)	0.321		
Pathological grade (high vs. low)	1.34 (0.78–2.11)	0.192		
circRNA CCT3 expression (high vs. low)	2.03 (1.35–2.99)	0.001*	1.92 (1.22–2.63)	0.001*

### Inhibition of circRNA CCT3 depresses the growth of BCa cells

**Fig. 4:**
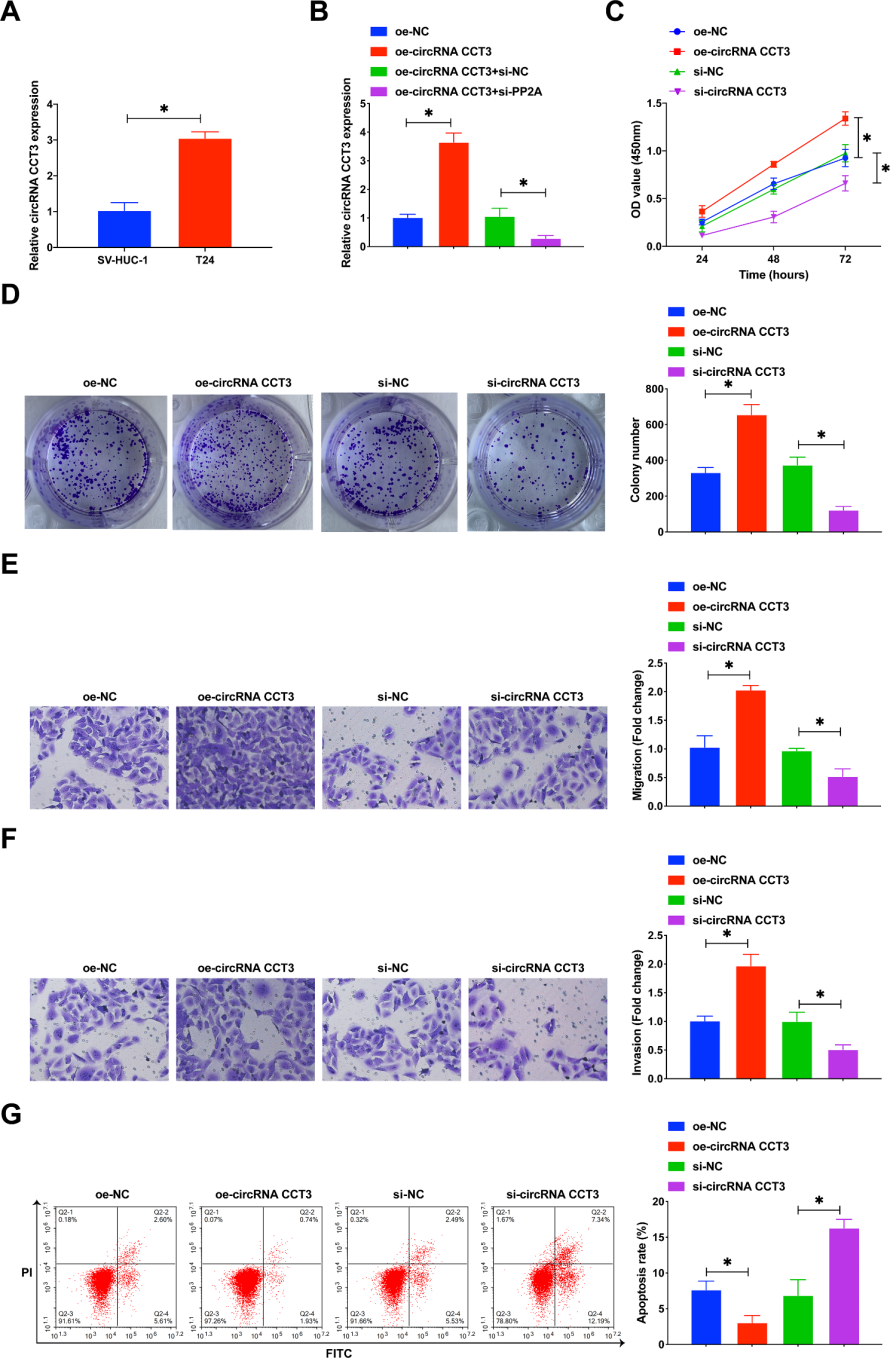
Inhibition of circRNA CCT3 depresses the growth of BCa cells. **(A)** RT-qPCR measured circRNA CCT3 in SV-HUC-1 cells and T24 cells. **(B)** RT-qPCR measured circRNA CCT3 in T24 cells transfected with circRNA CCT3 overexpression plasmid or si-circRNA CCT3. **(C)** CCK-8 assay detected cell proliferation rate. **(D)** Colony formation assay detected cell proliferation capacity. E/F. Transwell assay detected cell invasion and migration. G. Flow cytometry detected apoptosis rate. Data are expressed as mean ± SD (N = 3). * *P* < 0.05

Next, circRNA CCT3 in BCa tumor progression was further investigated in cells. Consistent with online database analysis, circRNA CCT3 expression was upregulated in T24 cell line compared to SV-HUC-1 cell line (Fig. 4A). Subsequently, siRNA and overexpressed plasmids targeting circRNA CCT3 were transfected into T24 cells. qPCR results confirmed successful knockdown and overexpression of circRNA CCT3 (Fig. 4B). CCK-8 and colony formation experiments revealed that overexpressed circRNA CCT3 further increased the proliferation rate and number of cloned cells in T24 cells, while knocking down circRNA CCT3 repressed T24 cell proliferation (Fig. 4C, D). Transwell assay results indicated that overexpressed circRNA CCT3 forced cell migratory and invasive capacities, while downregulated circRNA CCT3 did the opposite (Fig. 4E, F). Also, flow cytometry showed the effect of circRNA CCT3 on apoptosis of T24 cells. Elevating circRNA CCT3 increased the apoptosis rate of T24 cells, while knocking down circRNA CCT3 enhanced cellular apoptosis (Fig. 4G). In conclusion, inhibiting circRNA CCT3 reduced the malignant progression of BCa cells.

### circRNA CCT3 regulates PP2A expression by miR-135a-5p

**Fig. 5:**
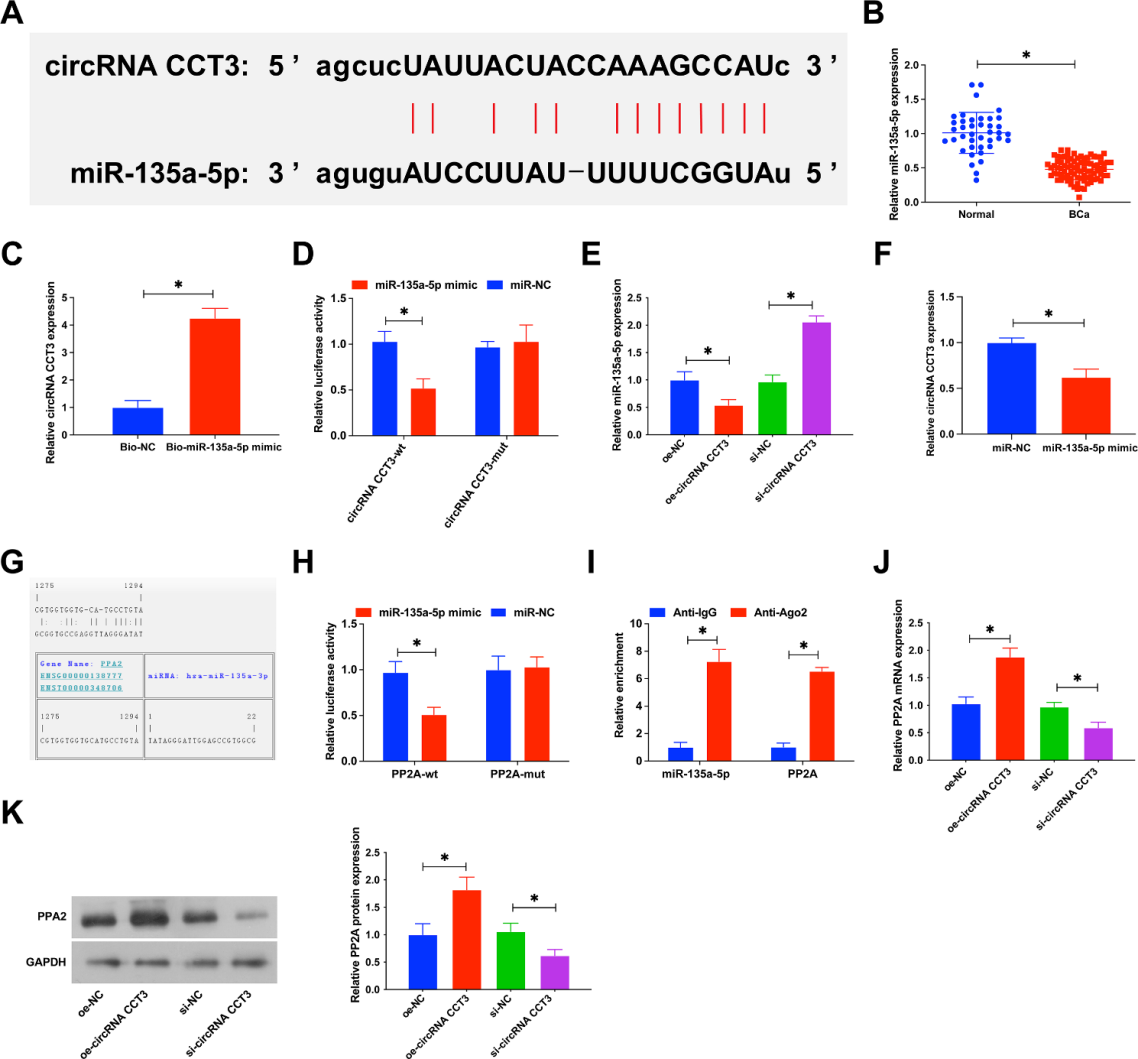
circRNA CCT3 regulates PP2A expression by miR-135a-5p. **(A)** The bioinformatics website predicts the binding site of circRNA CCT3 and miR-135a-5p. **(B)** miR-135a-5p expression in BCa tissue. **C-D**. Binding of circRNA CCT3 and miR-135a-5p. **E**. miR-135a-5p expression after regulating circRNA CCT3. **F**. Effect of overexpression of miR-135a-5p on circRNA CCT3 expression. **G-I**. Binding relation of miR-135a-5p and PP2A; **J-K**. PP2A expression after regulating circRNA CCT3.

Predicted results from the starBase database found that circRNA CCT3 may bind to miR-135a-5p in BCa cells. Complementary sequences between circRNA CCT3 and miR-135a-5p were potential binding sites (Fig. 5A). miR-135a-5p in BCa tissues was lower than that in normal tissues as measured by RT-qPCR (Fig. 5B). Results of RNA pull-down assay showed that miR-135a-5p was targeted to circRNA CCT3 (Fig. 5C). Dual luciferase reporter experiment indicated that co-transfection of miR-135a-5p mimic and circRNA CCT3-WT could reduce luciferase activity (Fig. 5D). Subsequently, the effect of circRNA CCT3 on miR-135a-5p was detected, and RT-qPCR results reported that overexpressed circRNA CCT3 lowered miR-135a-5p expression in BCa cells, while silencing circRNA CCT3 enhanced miR-135a-5p levels (Fig. 5E). In addition, overexpression of miR-135a-5p significantly reduced circRNA CCT3 expression (Fig. 5F). These data indicate that miR-135a-5p is the downstream miRNA of circRNA CCT3. Subsequently, the potential candidate target mRNA of miR-135a-5p was further investigated. miR-135a-5p and PP2A 3’UTR had potential binding sites (Fig. 5G). Subsequent dual luciferase reporter assay confirmed this prediction, showing that co-transfection with PP2A-WT and miR-135a-5p mimic could reduce luciferase activity (Fig. 5H). Ago2 RIP analysis was performed at the same time to verify the combination of the two. Compared with IgG group, miR-135a-5p and PP2A were specifically enriched on the same AgO2-based complex (Fig. 5I). To study the regulation of circRNA CCT3 on PP2A, qPCR and immunoblotting detected PP2A after regulating circRNA CCT3. The results discovered that overexpressed circRNA CCT3 increased PP2A, while silenced circRNA CCT3 forced PP2A levels (Fig. 5J, K). The above experiments indicated that circRNA CCT3 acted as a sponge for miR-135a-5p to regulate PP2A.

### circRNA CCT3 promotes malignant behavior of BCa cells by regulating the miR-135a-5p/PP2A axis

**Fig. 6:**
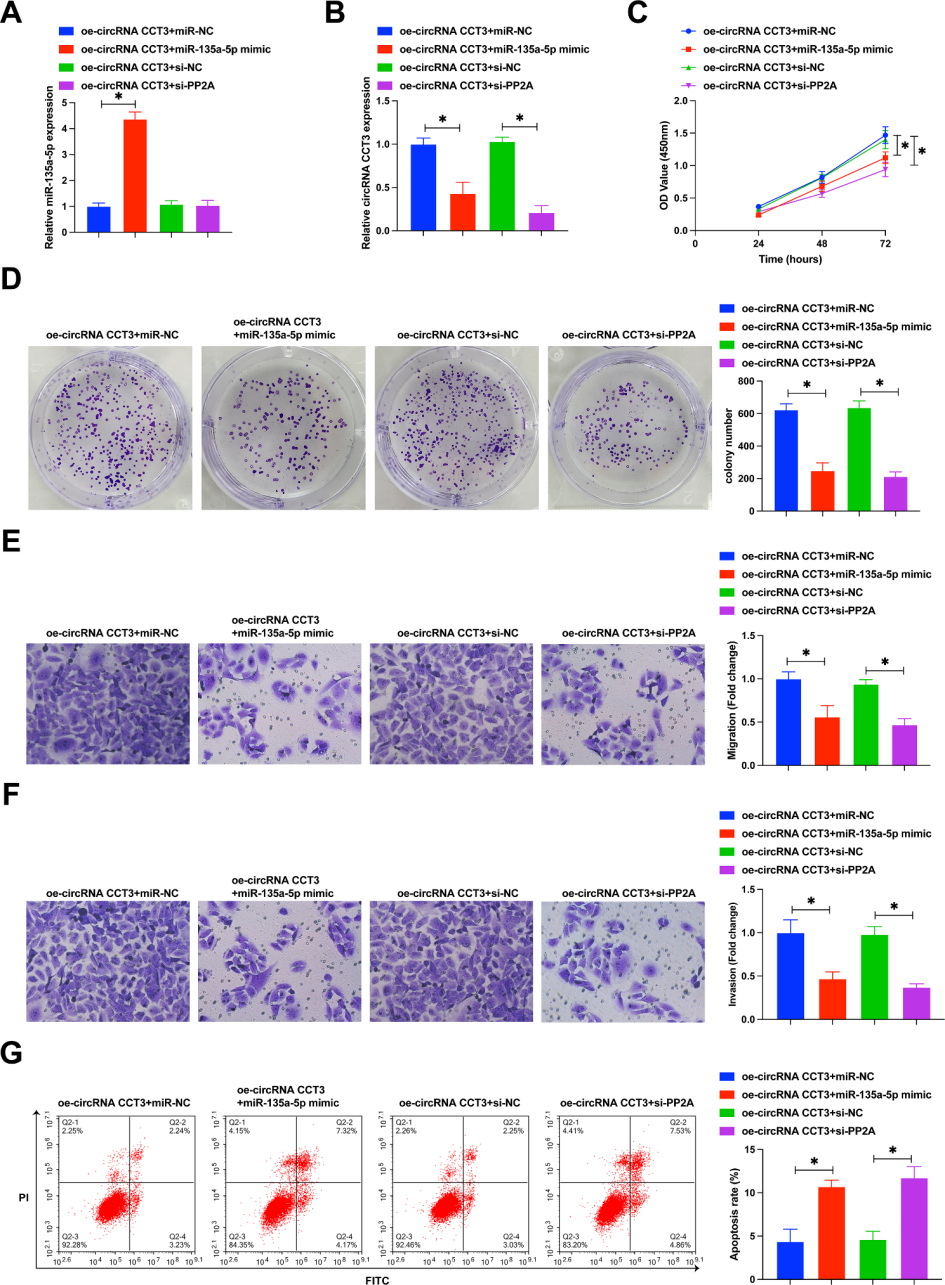
circRNA CCT3 promotes the malignant behavior of BCa cells by regulating the miR-135a-5p/PP2A axis. **(A)** RT-qPCR measured miR-135a-5p. **(B)** RT-qPCR measured PP2A. **(C)** CCK-8 assay detected cell proliferation rate. (D) Colony formation assay detected cell proliferation capacity. E/F. Transwell assay detected cell invasion and migration. G. Flow cytometry detected apoptosis rate. Data are expressed as mean ± SD (N = 3). * *P* < 0.05

The role of miR-135a-5p/PP2A axis in the regulation of BCa by circRNA CCT3 was analyzed through functional rescue experiments. Overexpression of circRNA CCT3 was accompanied by overexpression of miR-135a-5p or knockdown of PP2A, respectively. RT-qPCR experiments showed that overexpression of circRNA CCT3 inhibited miR-135a-5p and promoted PP2A expression, but this effect was reversed by overexpression of miR-135a-5p or knockdown of PP2A, respectively (Fig. 6A, B). CCK-8 and colony formation experiments showed that the promotion effect of circRNA CCT3 on the proliferation of T24 cells was reversed by overexpression of miR-135a-5p or PP2A knockdown (Fig. 6C, D). Transwell experiments showed that overexpression of circRNA CCT3 could promote the invasion and migration ability of T24 cells, but overexpression of miR-135a-5p or PP2A knockdown could reverse this phenomenon (Fig. 6E, F). In addition, the inhibitory effect of overexpression of circRNA CCT3 on the apoptosis rate of T24 cells was reversed by overexpression of miR-135a-5p or PP2A knockdown (Fig. 6G). These data suggest that circRNA CCT3 affects the malignant behavior of BCa cells by regulating the miR-135a-5p/PP2A axis.

## Discussion

BCa is one of the most common tumors of the urinary system, with high morbidity and mortality worldwide [28]. The prognosis of patients with advanced BCa is poor, so early diagnosis and the search for reliable and effective therapeutic targets are needed [29]. Many circRNAs have been shown to be abnormally expressed in BCa, and they are not only involved in regulating various biological behaviors of BCa, but also some circRNAs can be biomarkers for the diagnosis of BCa [30, 31]. However, the current understanding of circRNA as a biomarker and cancer factor for the early diagnosis of BCa is not comprehensive. This study analyzed clinical samples from BCa patients and screened out significant differentially expressed circRNA molecules. Among these abnormally expressed circRNAs, circRNA CCT3 (hsa_circ_0004680) was particularly studied. It was highly expressed in BCa and mediated BCa cell activities via miR-135a-5p/CCT3 axis. In addition, circRNA CCT3 in plasma was found to be a potential biomarker for BCa diagnosis.

Diagnostic biomarkers are of great significance for the early screening and malignant differentiation of BCa [32]. Since blood samples are most readily available to patients during early screening, blood biomarkers are important for the diagnosis of BCa [33]. Although many circRNAs have been confirmed to be involved in various biological behaviors of BCa cells, few can be considered diagnostic biomarkers. This may be due to the large abnormalities in the abundance of different circRNAs in cancer tissues and blood. This study found that circRNA CCT3 was highly expressed and had a high abundance in both BCa tissues and blood of BCa patients. High levels of circRNA CCT3 were confirmed by ROC curve as a potential diagnostic biomarker for BCa. Although previous studies have confirmed abnormal expression of circRNA CCT3 and its involvement in cancer development [34, 35], this study is the first to demonstrate the potential value of circRNA CCT3 in cancer diagnosis. Furthermore, circRNA CCT3 was associated with poor outcomes in BCa patients. The reason can be found in cell experiments, demonstrating that overexpressed circRNA CCT3 can promote proliferative, invasive, and migratory capacities of BCa cells and inhibit apoptotic capacity. It is speculated that high levels of circRNA CCT3 will accelerate the deterioration of BCa and thus reduce the survival time of patients with BCa. Notably, two previous studies have also shown that circRNA CCT3 promotes distal metastasis of cancer cells and EMT [16, 34]. It is speculated that circRNA CCT3 may also have similar biological functions in BCa, but it needs to be verified in subsequent xenotransplantation modelsSubsequently, the dual luciferase reporter assay, RNA-pull down assay, and RIP assay confirmed that circRNA CCT3 mediated PP2A by competitive adsorption of miR-135a-5p. It is speculated that the miR-135a-5p/PP2A axis may be one of the downstream pathways that circRNA CCT3 plays the role of oncogene in BCa. miR-135a-5p acts as a cancer suppressant in various cancers [20, 36, 37]. This study confirmed that miR-135a-5p is lowly expressed in BCa, but it is not clear whether it is a cancer suppressor in BCa, which needs to be verified in future studies. In addition, it is necessary to explore the role of circRNA CCT3/miR-135a-5p/PP2A in the biological behavior of BCa through functional rescue studies. This will provide data support for the search of potential therapeutic targets for BCa.

## Conclusion

In conclusion, this study demonstrated the specific high expression of circRNA CCT3 in BCa. circRNA CCT3 expression is closely related to the malignancy of BCa and the prognosis of BCa patients. These results suggest that circRNA CCT3 has a potential value in the diagnosis and treatment of BCa as a unique molecular marker. In addition, signaling pathways associated with circRNA CCT3 in BCa are discovered, which provide new ideas and directions for further research and treatment of BCa.

### Electronic supplementary material

Below is the link to the electronic supplementary material.

Supplementary Material 1

Supplementary Material 2

## Data Availability

The data and materials used to support the findings of this study are available from the corresponding author.

## Abbreviations

BCa: Bladder cancer
circRNA CCT3: Circular RNA chaperonin containing TCP1 subunit 3
RT-qPCR: Reverse transcription-quantitative polymerase chain reaction
ROC: Receiver operating characteristic
AUC: Area under the curve
PP2A: Protein phosphatase 2A
miR: MicroRNA
MIBC: Muscle-invasive BCa
circTAF4B: Circular RNA TAF4B
EMT: Epithelial-mesenchymal transition
CircSMARCA5: Circular RNA SMARCA5
OS: Overall survival
FBS: Fetal bovine serum
CCK-8: Cell counting kit-8
DMEM: Dulbecco’s Modified Eagle’s Medium
FITC: Fluorescein isothiocyanate
GAPDH: Glyceraldehyde-3-phosphatede hydrogenase
RIPA: Radioimmunoprecipitation assay
PVDF: Polyvinylidene fluoride
SDS-PAGE: Sodium dodecyl sulfate-polyacrylamide gel electrophoresis
ECL: Enhanced chemiluminescence
RIP: RNA immunoprecipitation
wt: Wild-type
mut: Mutant type
ANOVA: Analysis of variance
RFS: Recurrence-free survival
LNM: Lymph node metastasis
CI: Confidence interval
circMYLK: Circular RNA myosin light chain kinase
circPPP1CB: Circular RNA PPP1CB
SMG1: Suppressor with morphogenetic effect on genitalia family member
NR4A3: Nuclear receptor subfamily 4 group A member 3
