# Supplementary figures and images for: Circular RNA CCT3 is a unique molecular marker in bladder cancer
Source: BMC Cancer. 2023 Oct 13;23:977. doi: 10.1186/s12885-023-11510-0 (PMC10571266; doi:10.1186/s12885-023-11510-0)

GAPDH

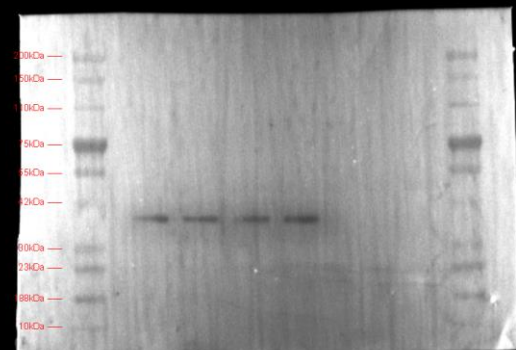

GAPDH 2

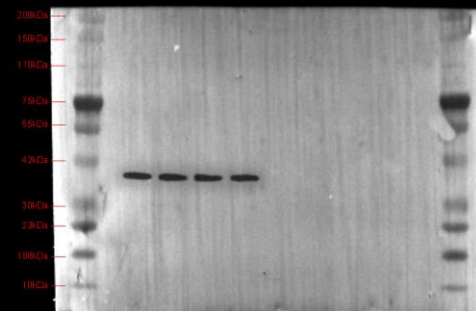

GAPDH 3

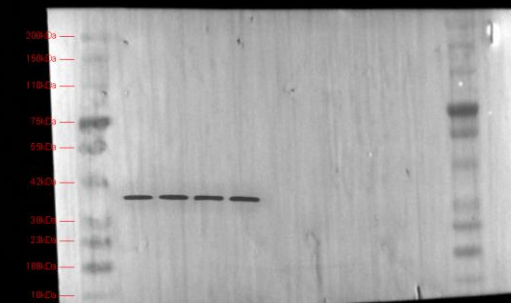

PP2A

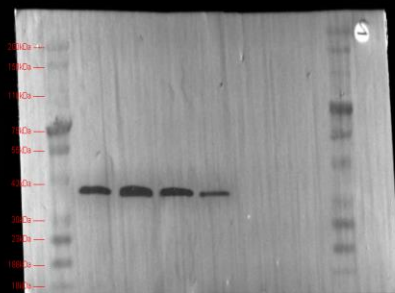

PP2A 1

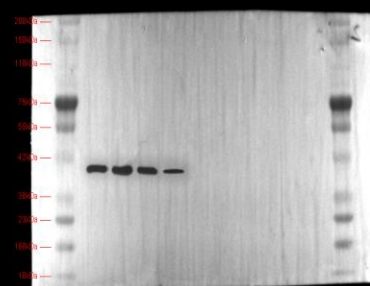

PP2A 2

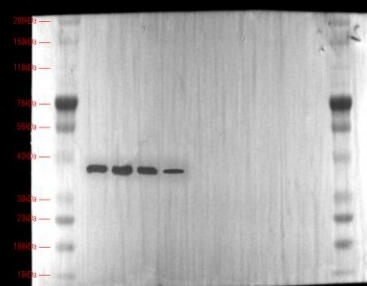

PP2A 3

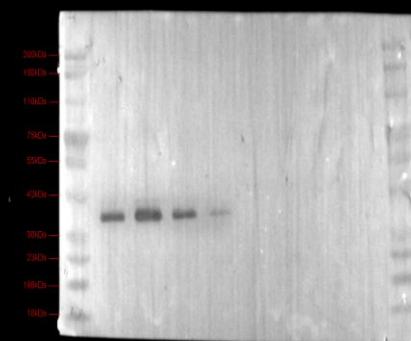

Supplement: Supplementary file 1 — Supplementary Material 1 [file 12885_2023_11510_MOESM1_ESM.pdf]
